# Elevated CSF angiopoietin-2 correlates with blood-brain barrier leakiness and markers of neuronal injury in early Alzheimer’s disease

**DOI:** 10.21203/rs.3.rs-2722280/v1

**Published:** 2023-04-18

**Authors:** James Miners, Carol van Hulle, Selvi Ince, Erin Jonaitis, OC Okonkwo, Barbara Bendlin, Sterling Johnson, Cynthia Carlsson, Sanjay Asthana, Seth Love, Kaj Blennow, Henrik Zetterberg

**Affiliations:** University of Bristol; Wisconsin Alzheimer’s Institute, University of Wisconsin School of Medicine and Public Health; Wisconsin Alzheimer’s Institute, University of Wisconsin School of Medicine and Public Health; Wisconsin Alzheimer’s Institute, University of Wisconsin School of Medicine and Public Health; Wisconsin Alzheimer’s Institute, University of Wisconsin School of Medicine and Public Health; Wisconsin Alzheimer’s Institute, University of Wisconsin School of Medicine and Public Health; Wisconsin Alzheimer’s Institute, University of Wisconsin School of Medicine and Public Health; UW; UW; University of Gothenburg; University of Gothenburg; University of Gothenburg

## Abstract

Breakdown of the neurovascular unit in early Alzheimer’s disease (AD) leads to leakiness of the blood-brain barrier (BBB), contributing to cognitive decline and disease pathology. Vascular stability depends on angiopoietin-1 (ANGPT1) signalling, antagonised by angiopoietin-2 (ANGPT2) upon endothelial injury. We have examined the relationship between CSF ANGPT2 and CSF markers of BBB leakiness and disease pathology, across three independent cohorts: (i) 31 AD patients and 33 healthy controls grouped according to their biomarker profile (i.e., AD cases t-tau > 400 pg/mL, p-tau > 60 pg/mL and Aβ42 < 550 pg/mL); (ii) 121 participants in the Wisconsin Registry for Alzheimer’s Prevention or Wisconsin Alzheimer’s Disease Research study (84 participants cognitively unimpaired (CU) enriched for a parental history of AD, 19 participants with mild cognitive impairment (MCI), and 21 with AD); (iii) a neurologically normal cohort aged 23–78 years with paired CSF and serum samples. CSF ANGPT2 level was measured by sandwich ELISA. In cohort (i), CSF ANGPT2 was elevated in AD, correlating with CSF t-tau and p-tau181 but not Aβ42. ANGPT2 also correlated positively with CSF sPDGFRβ and fibrinogen – markers of pericyte injury and BBB leakiness. In cohort (ii), CSF ANGPT2 was highest in MCI. CSF ANGT2 correlated with CSF albumin in the CU and MCI cohorts but not in AD. ANGPT2 also correlated with t-tau and p-tau and with markers of neuronal injury (neurogranin and α-synuclein) and neuroinflammation (GFAP and YKL-40). In cohort (iii), CSF ANGPT2 correlated strongly with the CSF:serum albumin ratio. Increased CSF ANGPT2 and the CSF:serum albumin ratio showed non-significant associations with elevated serum ANGPT2 in this small cohort. Together, these data indicate that CSF ANGPT2 is associated with BBB leakiness in early AD and is closely related to tau pathology and neuronal injury. The utility of serum ANGPT2 as a biomarker of BBB damage in AD requires further study.

## Introduction

Vascular pathology and dysfunction are demonstrable in most patients with Alzheimer’s disease (AD), which shares common risk factors with cerebrovascular disease (reviewed (1)). Neurovascular uncoupling and leakiness of the blood–brain barrier (BBB) contribute to cognitive decline and AD pathology (reviewed (2, 3)). In an imaging study of people with pre-clinical AD (i.e., having a clinical dementia rating of 0.5), BBB leakiness within the hippocampus was related to elevated CSF soluble platelet-derived growth factor receptor β (sPDGFRβ), a marker of pericyte injury. We previously reported that CSF sPDGFRβ level was elevated and correlated with CSF t-tau and p-tau levels in clinical AD confirmed by CSF biomarkers (i.e., t-tau > 400 pg/mL, p-tau > 60 pg/mL and Aβ42 < 550 pg/mL)(4). CSF sPDGFRβ was positively related to CSF t-tau and p-tau in two independent cohorts of cognitively unimpaired participants with biomarker changes spanning the spectrum from normal ageing to early AD (5, 6). CSF sPDGFRβ also correlated with PET-tau signal, and both markers were inversely related to cerebral blood flow, the associations being stronger in PET Aβ-positive individuals (7). A recent study found that sPDGFRβ level was highest in MCI and was elevated in MCI-converters compared to MCI patients whose cognitive performance was stable over a 1-year period (5). Together, the data point to microvessel-tau interactions that are associated with BBB leakiness and reduced blood flow, and which are probably exacerbated by the deposition of Aβ.

Angiopoietin (ANGPT) signalling via tyrosine kinase with immunoglobulin-like and EGF-like domains 1 and 2 (TIE-1 and TIE-2) receptors on endothelial cells, regulates vascular stability and BBB permeability in adult tissues (8, 9). ANGPT1, released by pericytes, activates TIE-2 receptors on endothelial cells, mediating vascular stability and BBB integrity. In contrast, ANGPT2, released predominantly by endothelial cells in response to injury, acts as a weak agonist or an antagonist of TIE-2, and is associated with angiogenesis (10, 11) and BBB leakiness (8, 12). ANGPT2 is upregulated in endothelial cells in response to hypoxia, via a hypoxia-inducible factor (HIF)1α-dependent mechanism (13). Recombinant ANGPT2 induced BBB leakiness, potentially via endothelial apoptosis, in a cortical cold-injury rat model (12). Infarct size and BBB permeability after transient middle cerebral artery occlusion in an ANGPT2 gain-of-function mouse model, were reversed on restoration of Tie-2 signalling (8). ANGPT2 levels were raised in the vitreous humour in patients with diabetic retinopathy (DR) (14) and chronically elevated in a rodent model of DR (15). In the rodent model, recombinant ANGPT2 triggered pericyte loss and vascular instability (15). ANGPT2 expression was elevated in microvessel-enriched preparations of brain tissue in AD (16), in which pericyte loss and BBB leakiness have been reported at an early disease stage (17–19) and was elevated concurrently with markers of angiogenesis in the cortex of young (2 month) J20 APP over-expressing mice (20).

We have explored the relationships between CSF ANGPT2; markers of pericyte injury (CSF sPDGFRβ) and BBB leakiness (CSF fibrinogen and albumin); established markers of AD pathology (CSF Aβ and tau); and markers of neuronal injury (neurogranin and α-synuclein) and neuroinflammation (GFAP and YKL-40), in three independent cohorts. The first cohort comprised AD and controls stratified according to CSF AD biomarkers; the second consisted predominantly of at-risk cognitively unimpaired (CU) controls, but also included individuals with mild cognitive impairment (MCI) and patients with established AD. In a third cohort, we have investigated the relationships between ANGPT2 in CSF and serum, and between ANGPT2 and the CSF:serum albumin ratio, in paired CSF and serum samples from neurologically normal adult donors.

## Methods

### Study cohorts

Cohort (i): CSF aliquots from clinical diagnostic CSF samples from 33 AD cases and 31 controls were kindly provided by the Clinical Neurochemistry Laboratory at Sahlgrenska University Hospital. Patients whose CSF had abnormal levels of AD biomarkers (t-tau > 400 pg/mL, p-tau > 60 pg/mL and Aβ42 < 550 pg/mL) were classified as having AD (21). CSF t-tau, p-tau181P, and Aβ42 had previously been measured using commercial ELISAs (INNOTEST, Fujirebio, Belgium). The demographics of the cohort including gender and age at which CSF was collected, are shown in Table 1a. Cognitive status and APOE genotype were not recorded in these individuals. The study complied with Swedish Biobank law (Biobanks in Medical Care Act) and was approved by the Ethical Committee at University of Gothenburg, Sweden.

Cohort (ii): CSF aliquots were provided from the Wisconsin Registry for Alzheimer’s Prevention Study and Wisconsin Alzheimer’s Disease Research Center (WISC cohort). WISC participants’ cognitive performance and functional status had been adjudicated by consensus conference. Diagnoses of MCI or dementia due to suspected AD were assigned based on National Institute on Aging-Alzheimer’s Association criteria (14,15), without reference to biomarkers. The WISC sample included donors with a clinical diagnosis of ‘cognitively unimpaired’ (CU; n = 84), mild-cognitive impairment (n = 21), and established AD (n = 17) at baseline. All WISC participants had baseline CSF obtained by lumbar puncture (LP). For this study, CU participants were selected if they had had serial LP sampling of CSF. The demographics, including sex distribution, APOE genotype, age at LP, and Aβ and tau status, are summarised in Table 1b. Markers of AD pathology (Aβ42, Aβ40, t-tau and p-tau181) and a panel of markers of neuronal injury and neuroinflammation had previously been measured using Roche NeuroToolKit^®^ immunoassays (Roche Diagnostics International Ltd, Switzerland).

Cohort (iii): Paired serum and CSF aliquots from neurologically normal controls (n= 23) spanning a wide age-range (23–84 years) were obtained from the Blennow/Zetterberg lab. The CSF:serum albumin ratio had previously been determined by an immunoturbidimetric albumin method (Elecsys, Roche Diagnostics, Penzberg, Germany). The demographics of the cohort, including sex and age at LP, are presented in Table 1c.

### ANGPT2 ELISA measurement in CSF and serum

ANGPT2 level was measured by ELISA (Quantikine kit, R & D systems, U.K.) according to the manufacturer’s instructions. CSF was diluted 2-fold and serum 5-fold in assay. Absorbance was read at 450 nM in a FLUOstar OPTIMA plate reader (BMG labtech, Aylesbury, UK). Measurements were made in duplicate for CSF, and in a single well for serum, the concentration of ANGPT2 was determined by interpolation against a standard curve generated by serially diluting recombinant ANGPT2 (3000–23.5 pg/ml). Results are expressed in pg/ml.

### Albumin ELISA measurement in CSF

CSF albumin level was measured in CSF samples from the WISC cohort, by commercial sandwich ELISA (Cat no 108788) (Abcam, Cambridge, UK) as in our previous study (4). CSF samples were diluted 1 in 2000 and measured in duplicate. Absorbance was read at 450 nM in a FLUOstar OPTIMA plate reader (BMG labtech, Aylesbury, UK) and albumin concentration was interpolated from a standard curve derived by serial dilution of recombinant human albumin (200–3.125 ng/mL). Results are expressed in ng/ml.

### Statistical analysis

ANGPT2 datasets were normally distributed. A single outlier was identified in the serum ANGPT2 measurements and was removed prior to analysis. Pearson’s test was used to assess linear correlations, which were calculated for all CSF biomarkers. Linear mixed-effects models with random intercepts, age-at-lumbar-puncture as the measure of time, and CSF vascular biomarker as the outcome were used to test associations with tau positivity (> 24.8 pg/mL), and cognitive status.

## Results

### CSF ANGPT2 is elevated in AD and correlates with markers of BBB leakiness.

**Figure 1:**
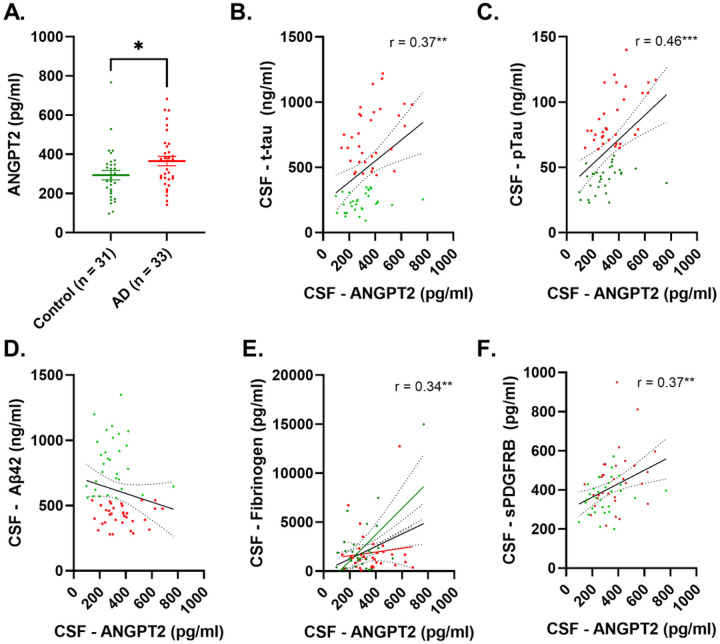
CSF level of ANGPT2 is elevated in AD in relation to CSF-tau and markers of BBB breakdown in Alzheimer’s disease. **A.** Dot blot showing significantly higher levels of ANGPT2 in Alzheimer’s disease (AD). **B-D** Scatterplot showing a positive correlation between ANGPT2 and t-tau and p-tau; no correlation was observed for Aβ42. **E-F** Scatterplots showing a positive correlation between CSF ANGPT2 level and CSF markers of BBB (fibrinogen and sPDGFRβ). In **A,** the bars represent the mean ± SEM. In **B-F**, the best-fit linear regression line is shown and 95% confidence intervals are superimposed. Each dot represents an individual sample. p < 0.05 was considered statistically significant

CSF ANGPT2 level was significantly higher in AD patients than controls (p < 0.05) (Fig. 1A). CSF ANGPT2 correlated positively with t-tau (r = 0.37, p < 0.01) and more strongly with p-tau (r = 0.46, p < 0.001) (Fig. 1B–C) but not with Aβ42 (r = −0.18, p = 0.15) (Fig. 1D). ANGPT2 correlated positively with CSF fibrinogen (r = 0.34, p < 0.01) and CSF sPDGFRβ (r = 0.37, p < 0.01) (Fig. 1E–F).

### CSF ANGPT2 is elevated in MCI and correlates with markers of BBB leakiness, neuronal injury and neuroinflammation.

**Figure 2:**
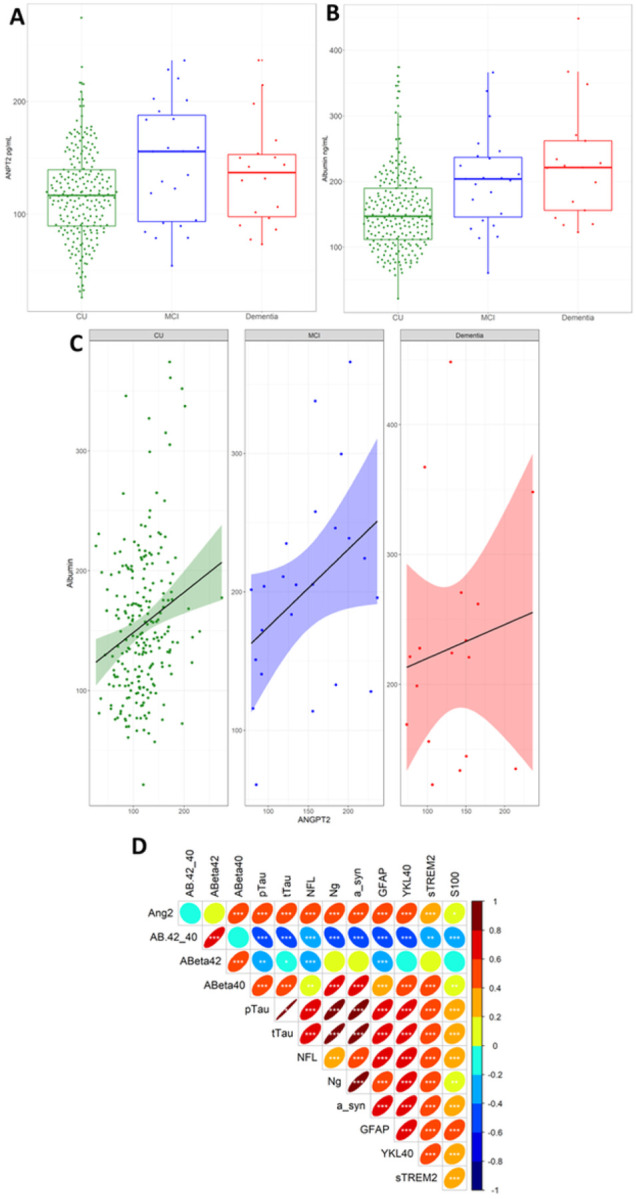
CSF ANGPT2 level is elevated in MCI and correlates with markers of BBB leakiness, neuronal injury and neuroinflammation. **A.** Boxplot showing elevated ANGPT2 in MCI (n = 21) compared to cognitively unimpaired (CU) controls (n = 81). ANGPT2 level was did not differ significantly between AD (n = 19) and CU controls. **B.** CSF albumin levels are higher in MCI (p = 0.068) and significantly higher in AD (p = 0.0009) compared to CU controls. **C.** CSF ANGPT2 is positively correlated with CSF albumin level in the CU and MCI groups but not in the AD group. **D.** A summary of correlations between CSF ANGPT2 and CSF levels of disease pathology (Aβ40, Aβ42, t-tau, p-tau181); neuronal injury (neurogranin (ng) and alpha-synuclein (a-syn, neuro lament light (nfl) and S100) and neuroinflammation (GFAP, sTREM-2 and S100). * p < 0.05, **p < 0.01, *** p < 0.001

CSF ANGPT2 was highest in MCI subjects and was significantly higher in MCI than cognitively unimpaired (CU) controls (p = 0.04) (Fig. 2A). Albumin level was raised in MCI (p = 0.068) and was significantly higher in AD than CU controls (p = 0.0006) (Fig. 2B). Among CU participants, CSF ANGPT2 but not albumin was associated with tau-positive status, i.e., CSF pTau181 > 24.8 pg/mL (p = 0.05 and p = 0.87 respectively). CSF ANGPT2 correlated with albumin in the CU (r = 0.21, p = 0.0008) and MCI groups (r = 0.43, p = 0.06) but not in AD cases (r = 0.09, p = 0.57) (Fig. 2C). CSF ANGPT2 correlated with sPDGFRβ across the entire cohort (r = 0.37, p = 0.0034).

CSF ANGPT2 level correlated positively with t-tau (r = 0.47, p < 0.0001) and p-tau (r = 0.45, p < 0.0001), and with Aβ40 (r = −0.44, p < 0.0001) but not Aβ42 (r = 0.15, p = 0.10). ANGPT2 correlated with markers of neuroinflammation – neurogranin (r = 0.46, p < 0.0001) and α-synuclein (r = 0.51, p < 0.0001); and markers of neuroinflammation – GFAP (r = 0.43, p < 0.0001) and YKL-40 (r = 0.42, p < 0.0001). A summary of the correlations between ANGPT2 and markers of AD pathology, neuronal injury and inflammation is shown in Fig. 2D.

### CSF ANGPT2 correlates with the CSF:serum albumin ratio in matched CSF and serum samples from neurologically normal controls.

**Figure 3:**
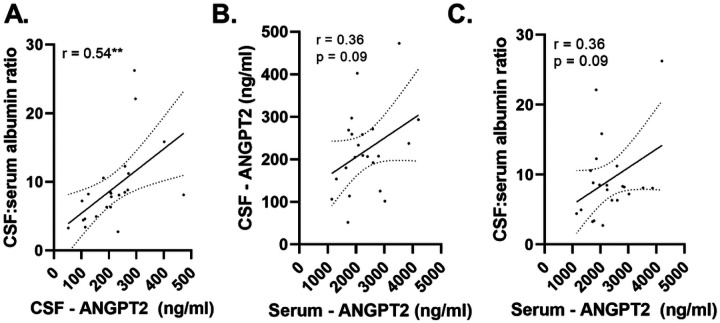
CSF and serum ANGPT2 correlated with CSF:serum albumin ratio. A scatterplot showing a positive correlation between CSF and serum ANGPT2 in matched serum and CSF samples. Scatterplots showing positive correlation between CSF and serum ANGPT2 and the CSF:serum albumin ratio. The best-fit linear regression line is shown and 95% confidence intervals are superimposed. Each dot represents an individual sample. p < 0.05 was considered statistically significant. ** p < 0.01.

CSF ANGPT2 level correlated strongly with the CSF:serum albumin ratio in matched CSF and serum samples from neurologically normal individuals (n = 23) (r = 0.54, p < 0.01) (Fig. 3 A). Elevated serum ANGPT2 tended to be associated with elevated CSF ANGPT2 (r = 0.36, p = 0.09), and with an elevated CSF:serum albumin ratio (r = 0.36, p = 0.097) but these relationships were not statistically significant (Fig. 3B– C).

## Discussion

In this study, we show that CSF ANGPT2 levels are elevated in AD and associated with BBB breakdown. ANGPT2 levels were raised in samples from individuals with CSF biomarker positivity for AD, based on established cut-off values for t-tau, p-tau and Aβ42 (21), and correlated with CSF t-tau and p-tau, and with markers of pericyte injury (sPDGFRβ) and BBB leakiness (CSF fibrinogen). Elevation of ANGPT2 is likely to occur early in the development of disease: in a cohort spanning the full spectrum of cognitive decline in AD, CSF ANGPT2 was highest in MCI and correlated most strongly with CSF albumin level in the cognitively unimpaired (CU) and MCI groups and not in AD, although CSF albumin levels continued to rise with disease progression. Across the same cohort, ANGPT2 correlated positively with CSF sPDGFRβ and with CSF t-tau and p-tau. CSF ANGPT2 also mirrored changes in CSF markers of neuroinflammation (YKL-40, GFAP and sTREM2) and neuronal injury (neurogranin and α-synuclein). In a third cohort comprising matched serum and CSF samples from healthy controls, CSF ANGPT2 correlated with the CSF:serum albumin ratio. Serum sPDGFRβ showed a trend towards positive correlation with both CSF ANGPT2 and the CSF:serum albumin ratio. Together, these data indicate that CSF ANGPT2 is a marker of pericyte injury and BBB leakiness, and its increase is associated with tau pathology, neuronal injury and cognitive decline in the early stages of AD. Whether, like CSF ANGPT2, serum ANGPT2 proves to be a useful indicator of BBB integrity will need to be determined in a larger study.

The ANGPT-Tie signalling pathway is a key regulator of vascular stability and is dysregulated in diseases including stroke and diabetic retinopathy, in which elevated ANGPT2 is associated with BBB leakiness and endothelial apoptosis (12, 22). Whether the ANGPT-Tie pathway is disrupted in AD is less well understood. ANGPT2 level was reported to be elevated in microvessels enriched from post-mortem AD brain tissue compared to healthy age-matched controls (16). ANGPT2 expression was elevated in young 2-month-old APP over-expressing J20 mice at an age when markers of pathological angiogenesis and increased vessel density were also observed suggesting that ANGP2 contributes to vascular instability in early AD (20). Our findings are in keeping with other evidence that damage to the cerebral vasculature occurs early in the development of AD. CSF ANGPT2 level was highest in MCI patients in whom it correlated with CSF albumin and sPDGFRβ, a marker of pericyte injury that was previously shown to be elevated in early AD (clinical dementia rating 0.5) and to correlate with MRI evidence of BBB breakdown within the hippocampus (17, 18). CSF ANPT2 level was strongly related to CSF t-tau and p-tau, as also reported for sPDGFRβ (4–7). A recent study revealed that sPDGFRβ correlated positively with the CSF:serum albumin ratio in patients with CDR 0–0.5 but not in established AD (5); the CSF:serum albumin ratio rose steadily with disease progression, as we found for CSF albumin in the present study. Mediation analysis revealed that BBB breakdown in the CDR 0–0.5 group reflected the influence of Aβ on pericyte degeneration, whilst BBB damage at a later disease stage was a direct effect of Aβ, by then presumably more abundant (5). Taken together with the present data, the findings suggest that abnormal ANGPT-Tie signalling is related to Aβ accumulation and tau pathology, and results in pericyte degeneration and BBB leakiness in early AD (7).

CSF markers of neuroinflammation and endothelial injury (ICAM-1, VCAM-1, YKL-40, IL-15 and VEGF-A) were reported to be elevated in the pre-clinical stages of AD and to be tightly associated with CSF tau, markers of cognitive decline, and cortical thinning – the relationship was strongest in individuals who were Aβ-positive on PET scan (23). In our study, CSF ANGPT2 level was strongly associated with CSF YKL-40 and GFAP, general markers of neuroinflammation, associated with astrogliosis in neurodegenerative conditions (24, 25). ANGPT2 was also moderately associated with CSF sTREM-2, which was previously reported to be elevated in MCI and strongly related to t-tau and p-tau but not Aβ42 (26). The authors of this last study suggested that the rise in CSF sTREM-2 reflected microglial activation in response to neuronal degeneration. We also found a strong correlation between CSF ANGPT2 and two additional CSF markers of neurodegeneration: neurogranin and α-synuclein. CSF neurogranin was reported to be elevated in AD in association with increased t-tau and p-tau (27), and to be strongly related to cognitive decline (28). CSF α-synuclein was also found to be raised in early AD and to be related to cognitive decline (29). ANGPT2 was recently shown to be elevated in cortical neurones in young 2-month old J20 mice with evidence of pathological angiogenesis and was induced in response to Aβ peptides in neural stem cells suggesting that Aβ-induced ANGPT2 expression within neurons contributes to vascular instability in early AD (20). Together, these data highlight the linkage between neuroinflammation, cerebral vascular injury, and neurodegeneration in AD.

CSF ANGPT2 levels do not correlate with CSF Aβ42; however, they are strongly correlated with CSF Aβ40. We and others previously reported a similar pattern of correlations for sPDGFRβ (4–6). CSF Aβ40 level is reduced in cerebral amyloid angiopathy (30), which is associated with accumulation of Aβ40 accumulation in cerebral blood vessels. Integrity of the BBB contributes to the clearance of toxic peptides within the brain. Pericytes internalise and clear Aβ peptides via LRP-1 mediated phagocytosis (31) and LRP-1 mediates the transcytosis of Aβ across the endothelium (32). The number of NG2-positive pericytes within the hippocampus was inversely related to the amount of guanidine-extracted insoluble Aβ40 (but not Aβ42) load (33) in human post-mortem brain tissue. Of probable relevance is the finding that fibrillar Aβ40 is toxic to pericytes in culture (33). The accumulation of Aβ40 may both contribute to and be exacerbated by the BBB leakiness and pericyte damage in AD.

We previously reported that serum and CSF sPDGFRβ levels correlated positively in paired serum and CSF samples healthy donors, but that serum sPDGFRβ was not related to the CSF:serum albumin ratio (4). In the present study, increased serum ANGPT2 tended to be associated with increased CSF ANGPT2 and an increased CSF:serum albumin ratio, although the correlation did not reach statistical significance. The utility of serum ANGPT2 as an indicator of BBB function will need to be determined through larger studies, preferably that also include MCI and AD patients; intriguingly, serum AGNPT1 level was previously been shown to be higher in AD than controls (34). If the ANGPT-Tie signalling pathway is deregulated in the early stages of AD, as suggested by our findings, this pathway would be a promising target for therapeutic intervention. The Tie-2 receptor agonist AV-001, which opposes the effects of ANGPT2, was shown to restore cognition in a rat model of multiple microinfarcts (35). Vaculotid, a Tie-2 agonist, also accelerated recovery following experimentally induced stroke in a rat model of diabetes (36). In a Phase II clinical trial, the bispecific antibody faricimab, a dual inhibitor of ANGPT2 and VEGF, improved visual acuity and reduced central subfield thickness in diabetic macular oedema (37).

In conclusion, CSF ANGPT2 appears to be a sensitive marker of pericyte injury and BBB breakdown in early AD. Its rise is closely related to tau pathology and neuronal degeneration, and also to neuroinflammation. The utility of serum ANGPT2 as a marker of pericyte injury and BBB breakdown merits further investigation.

## Figures and Tables

**Table 1a.** Summary of Cohort (i)

	Cases	Gender	Age at LP	CSF AB42	CSF t-tau	CSF p-tau
*Control*	n = 31	18M:12F	68.4 +/− 12.5	819.7 +/− 213.8	233.7 +/− 77.4	41.2 +/− 9.9
*AD*	n = 33	18M:15F	76 +/− 6.5	424.3 +/− 80.6	734.2 +/− 228.6	87.0 +/− 20.7

**Table 1b.** Summary of Cohort (ii)

	Ang2 (*N*=121)
**Sex**	
Female, n (%)	72 (59.5)
Male, n (%)	49 (40.5)
***APOE* ε4 carriership**	
*APOE*-, n (%)	60 (49.6)
*APOE*+, n (%)	61 (50.4)
**Age at LP**	
Mean (SD)	64.3 (6.79)
**Amyloid status**	
A-, n (%)	57 (48.3)
A+, n (%)	61 (51.7)
**Tau status**	
T-, n (%)	74 (62.7)
T+, n (%)	44 (37.3)
**Clinical Diagnosis**	
CU, n (%)	84 (69.4)
MCI, n (%)	20 (16.5)
Dementia, n (%)	17 (14.0)

*Abbreviations*: A+/− = Amyloid status, Ang2 = angiopoietin-2, APOE4 = apolipoprotein E ε4, CU = cognitively unimpaired, LP = lumbar puncture, MCI = mild cognitive impairment, sPDGFRβ = soluble platelet-derived growth factor receptor beta; T+/− = Tau status,

*Note*: Amyloid positivity was defined as CSF Aβ_42/40_ less than or equal to 0.046. Tau positivity was defined as CSF pTau_181_ concentration greater than 24.8 pg/mL. Clinical diagnosis was determined through a clinical consensus conference without reference to biomarker data.

**Table 1c.** Summary of cohort (iii)

Cases	Age +/− SD	Gender
*n = 23*	58.1 + 20.3	12M:11F
